# Supplementary material for: Modelling Skylarks (Alauda arvensis) to Predict Impacts of Changes in Land Management and Policy: Development and Testing of an Agent-Based Model
Source: PLoS One. 2013 Jun 6;8(6):e65803. doi: 10.1371/journal.pone.0065803 (PMC3675089; doi:10.1371/journal.pone.0065803)
Supplement: Supporting Information S4 — The skylark ODdox as a zipped archive. (ZIP) [file pone.0065803.s004.zip › Skylark_ODdox/functions_0x68.html]

ALMaSS Skylark ODdox: Class Members


ALMaSS Skylark ODdox
 2.0


- Main Page
- Related Pages
- Classes
- Files

- Class List
- Class Index
- Class Hierarchy
- Class Members

- All
- Functions
- Variables
- Typedefs

- a
- b
- c
- d
- e
- f
- g
- h
- i
- j
- k
- l
- m
- n
- o
- p
- r
- s
- t
- u
- v
- w
- x
- y
- z
- ~

Here is a list of all class members with links to the classes they belong to:

### - h -

- HandleEvents()
  : Farm
- Harvest()
  : Farm
- HasTramlines()
  : LE
- HaveSeen()
  : PolygonDataVector
- HaveTerritory
  : Skylark\_Male
- HayBailing()
  : Farm
- HayTurning()
  : Farm
- hb\_Add()
  : Landscape
- hb\_AddNewHedgebanks()
  : Landscape
- hb\_border\_pixels
  : Landscape
- hb\_Cleanup()
  : Landscape
- hb\_ClearPolygon()
  : Landscape
- hb\_core\_pixels
  : Landscape
- hb\_DownPolyNumbers()
  : Landscape
- hb\_FindBoundingBox()
  : Landscape
- hb\_FindHedges()
  : Landscape
- hb\_first\_free\_poly\_num
  : Landscape
- hb\_GenerateHBPolys()
  : Landscape
- hb\_HasNeighbourColor()
  : Landscape
- hb\_HasOtherNeighbour()
  : Landscape
- hb\_hedges
  : Landscape
- hb\_height
  : Landscape
- hb\_map
  : Landscape
- hb\_MapBorder()
  : Landscape
- hb\_MarkTheBresenhamWay()
  : Landscape
- hb\_MarkTopFromLocalMax()
  : Landscape
- hb\_max\_x
  : Landscape
- hb\_max\_y
  : Landscape
- hb\_MaxUnpaintedNegNeighbour()
  : Landscape
- hb\_min\_x
  : Landscape
- hb\_min\_y
  : Landscape
- hb\_new\_hbs
  : Landscape
- hb\_PaintBorder()
  : Landscape
- hb\_PaintWhoHasNeighbourColor()
  : Landscape
- hb\_ResetColorBits()
  : Landscape
- hb\_RestoreHedgeCore()
  : Landscape
- hb\_size
  : Landscape
- hb\_StripingDist()
  : Landscape
- hb\_UpPolyNumbers()
  : Landscape
- hb\_width
  : Landscape
- Heath()
  : Heath
- HedgeBank()
  : HedgeBank
- Hedges()
  : Hedges
- HerbicideTreat()
  : Farm
- HillingUp()
  : Farm


- Generated on Thu Jan 10 2013 13:15:39 for ALMaSS Skylark ODdox by
   1.8.1.1
